# Role of Vascular Liver Diseases in Hepatocellular Carcinoma Development

**DOI:** 10.3390/cancers17132060

**Published:** 2025-06-20

**Authors:** Lucia Giuli, Valeria De Gaetano, Giulia Venturini, Ersilia Arvonio, Marco Murgiano, Antonio Gasbarrini, Francesco Santopaolo, Francesca Romana Ponziani

**Affiliations:** 1 Liver Unit, Centro Malattie dell’Apparato Digerente (CEMAD), Medicina Interna e Gastroenterologia, Fondazione Policlinico Universitario Gemelli, Istituto di Ricovero e Cura a Carattere Scientifico (IRCCS), 00168 Rome, Italy; lucia.giuli@guest.policlinicogemelli.it (L.G.); valeria.degaetano01@icatt.it (V.D.G.); giulia.venturini02@icatt.it (G.V.); ersilia.arvonio01@icatt.it (E.A.); antonio.gasbarrini@unicatt.it (A.G.); francesco.santopaolo@policlinicogemelli.it (F.S.); 2 Dipartimento di Medicina e Chirurgia Traslazionale, Universitaà Cattolica del Sacro Cuore, 00168 Rome, Italy; marco.murgiano01@icatt.it

**Keywords:** vascular liver diseases, hepatocellular carcinoma, tumorigenesis, Budd–Chiari syndrome, Fontan-associated liver disease, congenital portosystemic shunts, cavernous transformation of the portal vein, porto-sinusoidal vascular disorder

## Abstract

Hepatocellular carcinoma (HCC) is a frequent complication of various liver diseases, occurring with or without underlying cirrhosis. While cirrhosis and chronic liver inflammation are well-established major drivers of hepatocarcinogenesis, HCC can also develop in patients with vascular liver diseases (VLDs), highlighting an alternative pathway of disease progression. Alterations in liver perfusion appear to underlie the process of carcinogenesis. However, the specific molecular mechanisms involved in this process as well as the clinical presentation and imaging features of HCC in the most common VLDs are still a matter of debate. This review aims to evaluate the available literature on the topic to provide a deeper comprehension and analysis of current knowledge about the relation between VLDs and HCC. Specifically, we investigate how HCC affects VLDs such as Budd–Chiari syndrome, Fontan-associated liver disease, congenital portosystemic shunts, cavernous transformation of the portal vein, and porto-sinusoidal vascular disorder. Exploring the pathogenetic mechanisms and diagnostic challenges in HCC related to VLDs may have important therapeutic implications, helping to define targeted treatments for this poorly understood medical entity.

## Simple Summary

Hepatocellular carcinoma (HCC) can develop even in the absence of cirrhosis, as observed in vascular liver diseases, suggesting that changes in liver perfusion may drive alternative mechanisms of carcinogenesis. The occurrence of HCC is possible in Budd–Chiari syndrome (BCS) and Fontan-associated liver disease (FALD), while it appears to be rare in other vascular conditions such as congenital portosystemic shunts (CPSS), cavernous transformation of the portal vein (CTPV), and porto-sinusoidal vascular disorder (PSVD). A better understanding of the link between vascular liver disease and HCC may improve diagnostic and therapeutic strategies for this rare but clinically relevant condition.

## 1. Introduction

Hepatocellular carcinoma (HCC) is the most common primary liver tumor and the third leading cause of cancer-related mortality worldwide, with an incidence that continues to rise [1]. Cirrhosis and chronic liver inflammation are the primary risk factors for HCC, with chronic hepatitis B (HBV) and C virus (HCV) infections, excessive alcohol consumption, and metabolic dysfunction-associated steatotic liver disease (MASLD) representing the most significant underlying conditions [2].

HCC has also been reported in the setting of vascular liver diseases (VLDs), suggesting that these conditions may contribute to hepatocarcinogenesis [3]. Unlike other chronic liver diseases, where inflammation, fibrosis, and ultimately cirrhosis are the major drivers of hepatocarcinogenesis, the development of hepatocellular nodules in VLDs appears to be primarily related to alterations in hepatic perfusion [4]. Specifically, the imbalance between portal venous and hepatic arterial blood flow—characteristic of VLDs—leads to increased hepatic arterial inflow and chronic hemodynamic changes, which may promote the formation of hepatocellular nodules and, eventually, HCC [5].

However, the molecular mechanisms underlying the association between altered hepatic perfusion and tumorigenesis remain poorly understood. Moreover, hepatocellular nodules arising in the context of VLDs often lack the typical radiological features observed in cirrhotic patients, thereby complicating noninvasive diagnostic approaches [6].

The aim of this study is to provide a comprehensive overview of the current evidence regarding the incidence, pathogenesis—including molecular mechanisms—and imaging characteristics of HCC in VLDs such as Budd–Chiari syndrome (BCS), Fontan-associated liver disease, congenital portosystemic shunts (CPSSs), cavernous transformation of the portal vein (CTPV), and porto-sinusoidal vascular disorder (PSVD). Gaining a deeper understanding of the pathogenetic links between VLDs and HCC, as well as optimizing the diagnostic pathway, is essential to improve early detection and guide tailored therapeutic strategies in this patient population.

## 2. Budd–Chiari Syndrome and Hepatocellular Carcinoma

BCS, also referred to as hepatic venous outflow tract obstruction (HVOTO), is a rare condition characterized by impaired blood flow through the hepatic veins (HVs) or the inferior vena cava (IVC) [7]. This paper focuses on primary BCS, defined as thrombosis within the hepatic outflow tract without external compression by space-occupying lesions or invasion by malignancy or parasites [7]. Both HV and IVC may be affected at different levels, often with asynchronous and progressive involvement. Obstruction of the hepatic outflow tract typically results in the development of intra- or extra-hepatic collateral circulation [8].

A recent pooled analysis estimated the annual prevalence of BCS at approximately 11 cases per million people [9], with slight differences in epidemiology between European and Asian countries [8]. The etiology of primary BCS includes a wide range of prothrombotic conditions, which often coexist. The most common is myeloproliferative neoplasms (MPNs) associated with JAK2 mutations (seen in 40% of BCS patients), followed by inherited disorders such as Factor V Leiden mutation (20%) and, less frequently, protein C and S deficiencies (7%). Acquired conditions include antiphospholipid syndrome (15%), Behçet’s disease (5%), and paroxysmal nocturnal hemoglobinuria (2%). Oral contraceptive use is reported in about 35% of cases but is usually associated with another underlying risk factor [8].

This variety of etiologies impacts both long-term outcomes and treatment decisions, as different causes may lead to thrombosis in different locations [8].

Vascular alterations in BCS lead to perfusion abnormalities and the formation of hepatic nodules, most of which are benign. Benign nodules in BCS are predominantly represented by focal nodular hyperplasia (FNH)-like regenerative nodules (RNs), with a reported prevalence of 36–44% [5,10]. These lesions arise in the setting of impaired hepatic venous outflow, decreased portal perfusion, and compensatory hepatic arterial hyperperfusion. They typically develop in areas with preserved venous drainage, where arterial neovascularization and hyperperfusion promote hepatocellular proliferation [11]. FNH-like RNs may increase in size and/or number over time, but they often regress spontaneously. Importantly, no evidence currently supports malignant transformation of these nodules [12]. Imaging characteristics of FNH-like RNs can be heterogeneous. On computed tomography (CT), they often exhibit arterial phase hyperenhancement with isodensity or mild hyperdensity during the portal venous phase. Occasionally, a hypodense perinodular rim may be present, complicating the differential diagnosis [12]. On magnetic resonance imaging (MRI) with gadolinium-based contrast agents, these nodules may mimic HCC due to homogeneous arterial phase enhancement followed by a so-called “pseudo-washout” in the portal phase [12]. However, hepatobiliary phase imaging can be helpful in differentiating these lesions from HCC and is therefore considered essential in the evaluation of hepatic nodules in BCS.

HCC is a well-known complication of BCS [13], although its prevalence and risk factors remain debated due to heterogeneity among cases. The 5-year cumulative incidence of HCC in BCS patients is estimated at approximately 4% [7]. In a recent cohort study of 302 BCS patients treated with endovascular intervention, the cumulative incidence of HCC at 1, 3, and 5 years was 0.3%, 4.7%, and 7.7%, respectively. Preoperative liver cirrhosis and postoperative restenosis were identified as independent risk factors [14]. These findings align with those of a population-based study by Wester et al., which found that HCC primarily developed in patients with established cirrhosis, whereas its incidence in non-cirrhotic BCS was extremely low [15]. Another study of 113 BCS patients by Li et al. identified IVC obstruction and stricture of the hepatic venous outflow tract as significant risk factors for HCC development in this population [16]. Consistent with these findings, a more recent study confirmed this association and further highlighted the role of long-segment IVC strictures as an additional risk factor for BCS-associated HCC [17]. This correlation can be explained by chronic hepatic venous outflow obstruction leading to persistent hepatic congestion, resulting in centrilobular necrosis, regeneration, fibrotic remodeling, and ultimately malignant transformation. Other risk factors investigated across studies include age, sex, factor V Leiden mutation [17,18], history of viral hepatitis [13], and elevated hepatic venous pressure gradient (HVPG) [19]. However, only HVPG showed a statistically significant association with HCC development in BCS patients.

BCS is linked to hepatic fibrosis through mechanisms involving ischemia, hepatocyte necrosis, and subsequent liver dysfunction. Hepatic venous outflow obstruction contributes to increased sinusoidal pressure, hepatic congestion, portal hypertension (PH), and ascites. These hemodynamic changes activate sinusoidal endothelial cells and hepatic stellate cells (HSCs), leading to fibrin deposition and fibrosis, particularly in the central lobular zones [20]. This process triggers compensatory liver regeneration, including arterial hyperperfusion and hypertrophy in regions with preserved venous drainage [21]. Such mechanisms drive hepatocellular proliferation and promote the formation of regenerative nodules and HCC [12]. Thus, the principal carcinogenic pathway in BCS-related HCC appears to involve chronic hepatic congestion, followed by the development of hepatic cirrhosis. Notably, BCS-associated HCC tends to be better differentiated than HBV-associated HCC and is often associated with a more favorable prognosis [22], supporting the idea of a distinct underlying pathogenic mechanism [23].

HCC in BCS displays heterogeneous imaging characteristics. Radiological assessment cannot rely on the standard criteria used for cirrhotic patients, as no pathognomonic imaging pattern can be identified on CT or MRI, particularly in the absence of cirrhosis [7]. Notably, approximately 25% of malignant nodules do not demonstrate washout in the portal or delayed phases, while about 33% of FNH-like nodules may show washout, rendering this feature unreliable for diagnostic purposes. Consequently, the Liver Imaging Reporting and Data System (LI-RADS) and AASLD/EASL non-invasive diagnostic criteria for HCC are not applicable in patients with BCS [24]. Rizzetto et al. highlighted the utility of various imaging features, including hepatobiliary phase assessment, in aiding differential diagnosis [12]. For this reason, it is important to complement imaging with serum alpha-fetoprotein (AFP) measurement, as a rapid increase in AFP levels is associated with HCC.

Routine surveillance in BCS patients should include imaging every six months. In the presence of atypical imaging findings, rising AFP levels, progressive increase in nodule size across two consecutive imaging studies, or other concerning features, a liver biopsy is recommended—especially if there are three or more nodules and/or any lesion exceeds 3 cm in diameter [3].

## 3. Fontan-Associated Liver Disease and Hepatocellular Carcinoma

FALD encompasses a spectrum of congestive hepatopathies that develop after Fontan surgery in patients with univentricular congenital heart disease. This condition ranges from liver fibrosis to cirrhosis and is associated with an increased risk of HCC.

Complex congenital cardiac anomalies—such as tricuspid or mitral atresia, or hypoplastic left or right heart syndromes—are not amenable to biventricular repair. Initial surgical approach includes the creation of a superior cavopulmonary connection (e.g., Norwood and Glenn procedures), in which venous return from the superior vena cava (SVC) is directed to the pulmonary arteries. Meanwhile, the single ventricle pumps mixed blood from the IVC and pulmonary veins into the systemic circulation. The Fontan procedure completes the total cavopulmonary connection by diverting blood from both the SVC and IVC directly to the pulmonary arteries, bypassing the subpulmonary ventricle [25].

As a consequence, venous pressure increases due to both precapillary factors (e.g., Fontan conduit stenosis, elevated pulmonary vascular resistance) and postcapillary factors (e.g., impaired ventricular function, atrioventricular valve dysfunction). Hemodynamic alterations in Fontan physiology—including the absence of pulsatile flow, chronically elevated central venous pressure (CVP), and persistently low cardiac output—result in multiorgan congestion. Additionally, ventilation–perfusion mismatch and the development of veno-venous collaterals contribute to chronic hypoxia, further exacerbating organ dysfunction [26].

While the Fontan procedure has significantly improved survival in patients with previously fatal conditions, the unique hemodynamic environment introduces long-term complications that require dedicated management through adolescence and adulthood.

Regarding hepatic involvement, chronic passive congestion driven by elevated CVP is the key pathophysiological mechanism leading to hepatomegaly, progressive liver fibrosis, and ultimately cirrhosis [27]. In Fontan circulation, HVs drain directly into the Fontan circuit, exposing the liver to persistent venous hypertension. This pressure is transmitted to the hepatic sinusoids and may impair portal inflow, compounding the effects of low cardiac output and resulting in ischemic injury [28,29,30].

Moreover, chronic hepatic congestion can promote thrombosis [31], which contributes to hepatic damage and fibrosis via thrombin-mediated activation of HSCs [20,32]. Notably, anticoagulants have been shown to reduce HSC activation and fibrosis progression in animal models [20,33].

Hypervascular hepatic nodules are common after Fontan surgery [27,34], typically located peripherally and associated with elevated right atrial pressure [35]. In a cohort of 27 Fontan patients, FNH was the predominant histologic diagnosis among those with hypervascular nodules. These findings support the hypothesis that hepatic venous hypertension and reduced portal venous inflow promote compensatory arterialization and hepatocellular proliferation [35], yielding imaging patterns similar to those seen in BCS [36].

In FALD, benign nodules frequently exhibit features overlapping with both large regenerative nodules and FNH, often termed “FNH-like” nodules. Typical features are proliferation of normal hepatocytes without a prominent central scar, lobulated by thin fibrous septa with a more or less apparent ductular reaction [37].

HCC is a relatively rare complication following Fontan procedure. In a cohort study of 122 Fontan patients, the incidence of HCC was 0.8% and 2.9% at 10- and 20-years post-surgery, respectively. Tumors were usually solitary, large (median diameter 47 mm) and associated with significantly reduced 25-year survival [38]. In 2020, a study cohort of 1620 surviving patients attested its prevalence at only 0.3% [39]; despite the small rate, the study reported a 100% mortality and a young age at the diagnosis. A meta-analysis including a total of 1320 patients reported a 7% cumulative incidence at 30 years post-Fontan, with negligible risk before 10 years post-surgery [40]. The presence of cirrhosis may increase the risk of HCC development [38,41,42], although cases have also been reported in the absence of cirrhosis [43]. However, compared with HCC due to HCV, Fontan-related HCC appears to occur earlier in the course of chronic liver disease [44]. Independent predictors of cirrhosis and HCC include elevated CVP and severe atrioventricular valve regurgitation [45]. Additional risk factors may include situs inversus [46], while anticoagulant therapy has been suggested as potentially protective. Serum alpha-fetoprotein (AFP) may aid in early diagnosis.

On imaging, typical HCC shows arterial phase hyperenhancement followed by mild, late washout on dynamic imaging. Suspicion should be raised for large peripheral masses, contour deformities, or interval changes in size or echotexture [26,37]. However, FNH-like nodules in cardiac cirrhosis may mimic HCC due to washout in the delayed phase in up to 10% of cases [26]. MRI with hepatobiliary-specific contrast agents is helpful for differentiation, as HCC typically appears hypointense in the hepatobiliary phase. Diffusion-weighted imaging (DWI) can also aid in distinguishing HCC from benign lesions [47]. For indeterminate nodules, contrast-enhanced ultrasound (CEUS) may provide additional diagnostic value [48,49].

It is important to note that, compared to biopsy, the LI-RADS may overestimate the probability of malignancy in FALD, and cannot be reliably applied in this context or in other vascular liver diseases [50,51].

Although formal guidelines are lacking, general experts consensus support HCC screening in FALD though regular liver imaging [37,52]. Based on current evidence and expert opinion, HCC surveillance might be initiated at least 10 years after completion of the Fontan procedure and strongly considered earlier in case of Fontan circulatory failure, even in the absence of cirrhosis [37]. Choice of imaging technique is still under debate; however, a practical approach that has been proposed is combination of serial ultrasound (every 6 months) with contrast enhanced imaging at baseline (10 years after surgery) and periodically thereafter (at least every 1–2 years) to monitor the full spectrum of liver nodules [37,53].

## 4. Congenital Porto-Systemic Shunts and Hepatocellular Carcinoma

CPSSs represent a group of rare vascular malformations in which intestinal blood flow bypasses the liver, either partially or completely, draining directly into the systemic circulation. These anomalies originate during embryogenesis of the portal and systemic venous systems and may be associated with other congenital abnormalities. Despite their anatomical complexity and heterogeneity—in terms of location, configuration, size, number of vessels involved, and type of communication—CPSSs are generally classified into two main categories: intrahepatic and extrahepatic shunts.

Extrahepatic CPSSs (CEPSs) can be further divided into two types based on the presence or absence of intrahepatic portal venous flow. In type 1 CEPS, the portal vein is absent, and all portal blood is diverted into the IVC, resulting in a complete lack of intrahepatic portal flow. In contrast, type 2 CEPS involves a partial, side-to-side communication that preserves some portal flow to the liver via a hypoplastic portal vein [54]. In both types, liver function is typically preserved, and the development of cirrhosis is not a common feature.

Clinical presentation is highly variable, ranging from asymptomatic cases detected incidentally on imaging to early and severe complications. The most common manifestations include hepatic encephalopathy, hepatopulmonary syndrome, pulmonary hypertension, and endocrine disturbances [55,56]. Diagnosis is typically established via Doppler ultrasound or cross-sectional imaging and confirmed by phlebography with occlusion testing.

Liver nodules are highly prevalent in this population [57,58], and their occurrence appears to be closely linked to the degree of portal flow deprivation [59]. A retrospective cohort study demonstrated that patients lacking intrahepatic portal flow had a significantly higher risk of developing both benign and malignant hepatic tumors compared to those with partial portal perfusion [60].

Most liver nodules are benign, with FNH, nodular regenerative hyperplasia (NRH), and hepatocellular adenoma (HCA) being the most common types. However, malignant lesions, including HCC and hepatoblastoma (HB), have also been described, particularly in the contest of CEPSs [61,62,63]. Notably, HCC in this setting can occur even in the absence of cirrhosis [64]. Radiological features, molecular profiles, and the risk of malignant transformation differ significantly from those of conventional hepatic tumors. Benign lesions such as HCA and FNH may present atypical imaging features and carry a higher risk of malignant transformation. Moreover, discrepancies between imaging and final histopathological diagnoses are common, often due to the co-existence of multiple, heterogeneous nodules within the same patient [65].

In the pathogenesis of NRH, it is hypothesized that diversion of portal blood into the systemic circulation leads to uneven hepatic perfusion. This triggers compensatory hepatic arterialization and regenerative hyperplasia in response to local ischemia and nutrient deficiency [66]. A similar mechanism has been proposed for HCC development: progressive loss of portal perfusion and increased arterial blood supply may promote neoplastic transformation. Indeed, CT imaging of evolving HCCs often reveals increasing attenuation on arteriography and decreasing enhancement on arterioportography, reflecting neovascularization and portal vein obliteration [67]. Additionally, it is speculated that chronic increase in hepatic arterial flow may induce hepatocyte dedifferentiation, thereby contributing to carcinogenesis [54]. Nevertheless, the exact mechanisms remain poorly understood, especially in non-cirrhotic patients with vascular abnormalities.

A case of FNH progressing to HCC in a patient with CEPSs has been reported [68], while malignant transformation of HCA appears associated with β-catenin activation or CTNNB1 mutations [69]. These observations underscore the importance of close surveillance of hepatic nodules in patients with CPSSs.

Liver imaging in this population is challenging due to portal deprivation and increased arterial supply. Contrast-enhanced MRI and ultrasound are the preferred imaging modalities with hepatobiliary contrast agents especially useful for baseline characterization of liver nodules. In general, shunt closure is recommended in CPSS patients with liver lesions. Experts recommend performing imaging every six months prior to shunt closure, with increased frequency (every three months) in cases of diagnostic uncertainty [56]. However, while shunt closure may lead to regression or disappearance of nodules, cases of HCC have been reported even long after closure [60].

If nodules change in morphology or enhancement patterns, or if radiologic features remain inconclusive, liver biopsy is warranted [56]. Therefore, until reliable risk stratification tools become available, lifelong imaging surveillance is strongly advised [56] (Table 1).

## 5. Cavernous Transformation of the Portal Vein and Hepatocellular Carcinoma

CTPV is a compensatory response to portal vein thrombosis (PVT) or obstruction, aimed at restoring portal blood flow to the liver through recanalization and/or the formation of collateral veins. Following portal vein obstruction—particularly complete extrahepatic portal vein obstruction (EHPVO)—fibroblasts remodel the thrombus into a firm, collagenous plug within which tortuous venous channels subsequently develop.

Obstruction of the main portal vein, its intrahepatic branches, and/or proximal segments of the splanchnic–mesenteric venous system leads to increased presinusoidal pressure, which causes retrograde blood flow into the splanchnic–mesenteric circulation. This results in PH and relative hepatic blood deprivation, primarily localized to the hepatic hilum [71]. A compensatory increase in hepatic arterial flow is observed, especially in the peripheral liver segments. These vascular changes may mimic chronic liver disease, presenting with hypertrophy of the caudate lobe and liver segment IV and atrophy of the left lateral segment and right hepatic lobe [72].

CTPV may remain clinically silent for long periods. Clinical manifestations are related to signs and symptoms of PH, with gastroesophageal variceal bleeding, splenomegaly, and thrombocytopenia. Additionally, portal biliopathy may develop, characterized by biliary tract fibrosis, impaired motility, and chronic cholestasis [73]. The overall mortality rate associated with CTPV is approximately 10% in both adults and children, often related to variceal bleeding [74].

CTPV can occur in both cirrhotic and non-cirrhotic patients. In cirrhotic individuals, PVT is associated with static portal blood flow secondary to PH, compounded by endothelial injury from intestinal infections or inflammatory responses to medical interventions. In non-cirrhotic patients, PVT is more commonly linked to systemic hypercoagulable states—either inherited or acquired—including latent MPNs, protein C or S deficiencies, and antiphospholipid syndrome. In children, CTPV may result from congenital vascular anomalies or perinatal events such as prior umbilical vein catheterization, abdominal infection, or trauma [75,76].

Liver nodules are generally uncommon in this setting. Case reports have predominantly described FNH-like lesions, which typically exhibit benign imaging characteristics on MRI and a stable clinical course [77,78,79]. However, HCA and HCC have also been reported, although they appear to be rare [80].

Interestingly, in patients with liver cirrhosis and portal cavernomatosis secondary to PVT, the presence of cavernous transformation has been associated with more favorable outcomes. These include a lower risk of HCC development, reduced incidence of hepatic decompensation, and improved 10-year survival rates compared to patients without cavernomatosis [81].

## 6. Porto-Sinusoidal Vascular Disorder and Hepatocellular Carcinoma

PSVD is a rare vascular liver condition marked by peculiar histological alterations in the small portal venules and sinusoids and the absence of cirrhosis on liver biopsy. Previously known as idiopathic non-cirrhotic portal hypertension (INCPH), both patients with and without PH are now included under this new definition and the coexistence of other chronic liver diseases—such as viral hepatitis or MASLD—no longer excludes the diagnosis of PSVD [82]. The specific histological lesions associated with PSVD comprehend obliterative portal venopathy (also referred to as portal vein stenosis), distinguished by a narrowing of the portal vein branches; incomplete septal fibrosis, described by incomplete and thin septa which delimit rudimentary nodules; and NRH, characterized by micro-nodularity of the liver in the absence of fibrosis [83]. Although these lesions are characteristic of PSVD, they are not universally present in all cases.

The exact pathogenesis of this condition remains unclear. It is thought to result from damage and obliteration of the intrahepatic portal vein branches, causing elevated resistance to portal blood flow and the development of a presinusoidal form of PH [83,84] Over half of affected individuals have an identifiable associated condition—such as exposure to certain drugs, autoimmune or hematological disorders, infections, or congenital diseases—which are believed to contribute to the vascular injury affecting small- to medium-sized portal veins [84,85].

PSVD patients with PH are typically asymptomatic until they experience a complication associated with PH. Variceal bleeding is the first manifestation in 20–40% of cases. Ascites occurs in 20–50% of patients during the course of the disease while PVT is observed in 30–40% of cases within five years of diagnosis [83,86].

Similar to the other VLD listed above, liver nodules can also occur in PSVD, although they are less frequent [3].

Generally, hepatocellular nodules in PSVD are benign, most of them being FNH and FNH-like lesions. These hyperplastic, reactive lesions likely arise from imbalanced regional blood flow, marked by reduced portal venous inflow and increased arterial blood supply [87]. Typical imaging features of FNH-like lesions in PSVD include arterial phase hyperenhancement without portal venous washout, along with hyperintensity in the hepatobiliary phase on MRI using hepatobiliary contrast agents [88].

HCC appears to be a rare complication of PSVD, as supported by multiple studies [82]. Several retrospective and prospective cohorts, including patient numbers ranging from 43 to 62 and median follow-up periods between 46 and 90 months, reported no cases of HCC [88,89]. In contrast, the largest retrospective study to date, conducted by Magaz et al., including 587 patients, reported only three cases of HCC (0.5%), with a median interval of 52 months from PSVD diagnosis to HCC occurrence [90]. Similarly, a separate cohort of 91 patients followed for a median of 37 months identified two cases of HCC. Notably, 38.5% of this cohort had coexisting liver diseases such as viral hepatitis or MASLD, known risk factors for HCC [91]. It remains unclear whether the patients who developed HCC also had these comorbidities, making it difficult to establish PSVD as a direct causal factor for hepatocarcinogenesis.

Due to the rarity of HCC in this patient population, the mechanisms underlying carcinogenesis remain poorly understood and represent an area of ongoing investigation [4,92].

In light of current evidence, routine HCC surveillance is not recommended for PSVD patients in the absence of other risk factors [87]. However, due to the high risk of PVT in PSVD—especially in patients with associated PH—regular imaging follow-up is warranted [93].

Future studies are essential to determine whether PSVD independently increases the risk of HCC and to further elucidate the molecular and hemodynamic mechanisms involved in this potential association.

## 7. Treatment Strategy of Hepatocellular Carcinoma in Vascular Liver Disease

The treatment of HCC includes a range of therapeutic options, which are determined primarily by tumor stage, hepatic functional reserve (assessed via the Child–Pugh score), and patient performance status [1,94]. However, studies specifically addressing HCC treatment in the context of VLDs remain limited, making it difficult to establish evidence-based, standardized protocols. In the rare clinical scenario of patients with both HCC and VLDs, conventional therapies for early stage HCC may still offer curative potential [16].

Most of the available literature focuses on diagnostic and therapeutic strategies for HCC in BCS. In these patients, if technically feasible, transarterial chemoembolization (TACE) remains a viable treatment option. In those eligible, curative resection or ablation also represent an effective treatment option [16]. Additionally, angioplasty or stent placement to reduce obstruction of the IVC or HV may lower sinusoidal pressure and mitigate carcinogenic stimuli [95]. Close surveillance of BCS patients allows for earlier detection and more effective management of neoplastic lesions. In selected cases, liver transplantation may also be considered [16]. Treatment options for HCC in Fontan patients are often limited. PH may contraindicate surgical resection, while local–regional therapies can be complicated by the presence of pacemakers (e.g., for RFA), abnormal vascular anatomy (e.g., for TACE), or extrahepatic shunting (e.g., for radioembolization). Systemic therapy requires a careful evaluation of cardiac function before using potentially cardiotoxic drugs, such as tyrosine kinase inhibitors. Liver transplantation—or combined heart–liver transplantation—must be carefully assessed based on cardiac function and institutional experience [96]. CPSSs represent a specific case in which locoregional therapies, such as TACE, are typically avoided due to the risk of hepatic failure in the absence of portal perfusion. Although favorable response rates may be observed due to the exclusive arterial supply, complications are more likely. Surgical decision—including timing and modality of resection—should be based on histologic, molecular, and anatomical findings and discussed within a multidisciplinary team, taking into account local expertise and available resources [56].

Several factors complicate therapeutic decision-making in these settings. Vascular obstruction, PH, splanchnic vein thrombosis, and concurrent use of anticoagulant or antiplatelet agents can all influence the safety and efficacy of oncologic treatments. Careful assessment of treatment-related side effects is therefore essential when selecting the most appropriate strategy.

Systemic therapy with atezolizumab–bevacizumab or lenvatinib is associated with both thrombotic and hemorrhagic risks, primarily due to inhibition of vascular endothelial growth factor (VEGF). Preventing esophageal variceal bleeding requires early identification of bleeding risk factors—such as grade II–III varices, splenomegaly, or increased spleen stiffness (a marker of portal hypertension). An additional challenge arises in patients on direct oral anticoagulants (DOACs), who have an elevated bleeding risk. Nevertheless, gastrointestinal bleeding usually occurs only in the presence of other risk factors (e.g., large varices or previous variceal bleeding), whereas the risk of non-gastrointestinal bleeding is generally elevated across all patients [97].

In patients undergoing treatment with atezolizumab–bevacizumab, prophylactic management of esophageal varices is recommended before initiating therapy. An upper gastrointestinal endoscopy (EGD) should be performed prior to treatment and repeated every 6–12 months thereafter. Early identification and control of varices reduces the risk of bleeding during treatment, supporting the safe use of this regimen—even in patients with portal hypertension and esophageal varices receiving anticoagulation for conditions such as splanchnic thrombosis [98].

Immune checkpoint inhibitors (ICIs), including atezolizumab, may also induce immune-mediated cardiovascular toxicities such as myocarditis and pericarditis. VEGF inhibitors are known to cause adverse effects including heart failure and hypertension. Thromboembolic events are more frequent in patients receiving combination therapy (e.g., atezolizumab–bevacizumab) compared to those on monotherapy, suggesting a synergistic effect. Importantly, thromboembolism-related mortality is increased in these patients. Proposed mechanisms include increased tissue factor expression, T-cell activation, reduced nitric oxide and prostacyclin production, and enhanced exposure of procoagulant phospholipids on subendothelial surfaces [99].

Elevated C-reactive protein (CRP) levels at the start of systemic therapy have been identified as a potential marker for thromboembolic risk. Monitoring CRP and other plasma biomarkers may allow for earlier detection and management of thrombotic complications, particularly in high-risk populations such as patients with VLDs [99].

## 8. Discussion

**Figure 1:**
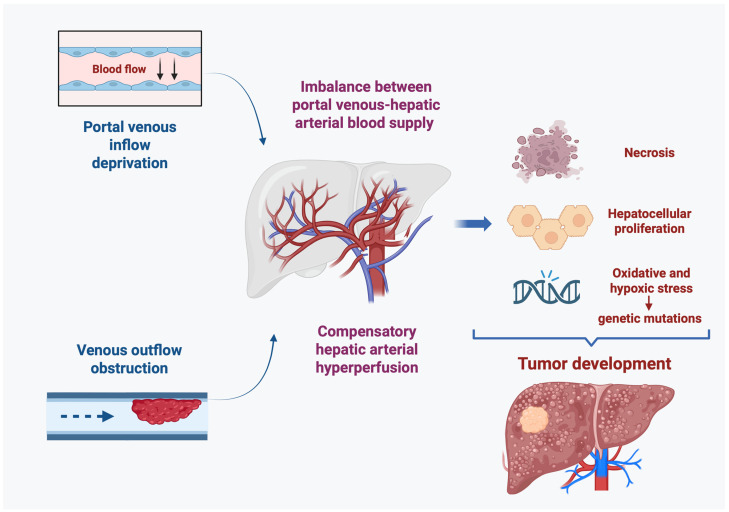
Tumorigenesis model in VLDs. Decreased portal perfusion either due to portal venous inflow deprivation or venous outflow obstruction is compensated by an increase in arterial blood flow. This compensatory hepatic arterial hyperperfusion is considered the trigger for the development of HCC in VLDs.

A wide spectrum of VLDs can lead to the development of hepatic nodules. Despite the heterogeneity of each disorder, a unifying pathogenic mechanism can be identified: either portal venous inflow deprivation or venous outflow obstruction results in an imbalance between portal venous and hepatic arterial blood supply [100] (Figure 1). Decreased portal perfusion triggers compensatory hepatic arterial hyperperfusion, which may play a role in tumor development, as shown in experimental models of portosystemic shunts [101]. Notably, portocaval shunts that preserved some degree of portal blood flow were associated with less necrosis, atrophy, and epithelial proliferation compared to complete shunts, emphasizing the role of optimal perfusion and hepatotropic factor delivery in preventing carcinogenesis [102].

While in BCS and in FALD the development of HCC is primarily driven by chronic hepatic congestion along with progressive hepatic injury and subsequent cirrhosis [16,18,22,103], in patients with CSSP, HCC can arise even in the absence of cirrhosis. In these cases, the complete deprivation of portal inflow appears to be the primary driver for tumoregenesis. Similarly, the compensatory cavernous transformation following PVT does not provide adequate blood flow to distal portions of the liver [3] (Table 2). In a pediatric population with extrahepatic portal vein obstruction who underwent portosystemic shunt surgery, 15% developed hepatic nodules after a median follow-up of 80 months [104], reinforcing this pathophysiological link.

The molecular pathways underlying HCC in VLDs remain poorly understood and warrant further investigations. Some evidence suggests that CTNNB1 mutations and β-catenin activation may contribute to malignant transformation in FNH-like nodules, even in the absence of underlying fibrosis [105]. This highlights a fundamental difference from sporadic FNH, as FNH-like lesions in VLDs may be carry neoplastic potential and a risk of malignant progression. However, it remains unclear whether Wnt signaling activation precedes nodule formation or is acquired secondarily.

In a murine model of chronic hepatic congestion obtained by partial IVC ligation, liver congestion was shown to promote HCC and metastatic liver tumor growth. Indeed, chronic hepatic congestion leads to increased portal pressure and intestinal permeability. Gut derived lipopolysaccharides (LPSs) subsequently induce capillarization and upregulation of Sphingosine Kinase 1 (SphK1) in liver sinusoidal endothelial cells (LSECs) [106]. Several studies have reported a close association between LSEC capillarization and both liver fibrosis and HCC [107,108]. Capillarized LSECs secrete sphingosine-1-phosphate (S1P), which plays a central role in both liver fibrosis and tumorigenesis in congestive hepatopathy. S1P activates HSCs via S1PR2, promoting liver fibrosis, and stimulates proliferation of the hepatocytes via S1PR1 [106]. Thus, modulation of S1P and LPS signaling could represent potential therapeutic targets to counteract liver fibrosis and hepatocarcinogenesis in chronic hepatic congestion. To further advance this field, future research should employ preclinical models that mimic chronic liver congestion and portal venous inflow deprivation. Understanding the molecular mechanism linking HCC to VLDs would be the first step towards developing tailored surveillance and treatment strategies. Omics-based approaches—including transcriptomics, proteomics, and genomics—could be particularly useful for delineating the molecular landscape of HCC in VLDs and identifying early biomarkers of malignant transformation.

Diagnosis of HCC in VLDs represents another open challenge. Differentiating benign from malignant hepatocellular nodules is particularly difficult due to the atypical imaging features frequently observed in VLDs [5]. MRI with hepatobiliary contrast agents can aid in distinguishing FNH-like lesions from HCC [109]. However, in the presence of atypical imaging characteristics—especially when associated with elevated serum AFP levels or nodule growth—a liver biopsy is recommended for definitive diagnosis [4]. These considerations underscore the need for careful surveillance using both imaging and serum biomarkers, as well as the role of liver biopsy in selected cases. A multidisciplinary team—including hepatologists, radiologists, and pathologists—is essential to guide the diagnostic process and ensure accurate characterization of liver nodules [6].

Treatment decisions must be individualized, taking into account the specific vascular abnormalities, the degree of PH, and the associated risk of complications. Locoregional therapies such as ablation, TACE, or hepatic resection may be effective when technically feasible. Liver transplantation may be considered in selected cases, with generally favorable outcomes [9]. Although systemic therapies with immune checkpoint inhibitors are approved and effective in cirrhotic patients, further studies are required to determine their safety and efficacy in patients with VLDs.

Overall, the management of HCC in this context remains a complex and evolving field that requires further investigation and dedicated clinical studies [16].

## 9. Conclusions

In conclusion, HCC can arise in the context of VLDs, and its pathogenesis is related to the imbalance between portal venous and hepatic arterial blood flow leading to an increased hepatic arterial inflow. However, the exact molecular mechanism involved in the pathogenesis of HCC in this setting requires further investigation. Diagnosis of HCC in VLDs poses a challenge because of the lack of typical radiological features, underlying the need for a complete work-out and the importance of a multidisciplinary team of expert guiding diagnosis and treatment decisions. Close surveillance and targeted therapy plan that consider the clinical condition of the patient and the underlying vascular abnormalities are crucial to improving outcomes in patients with VLDs. 

## Figures and Tables

**Table 1:** Hepatocellular carcinoma surveillance in vascular liver disease.

Vascular Liver Disease	HCC Risk	Surveillance Recommendation	Guideline Availability
Budd–Chiari Syndrome	Moderate	Abdominal ultrasound every 6 months ± AFP	Practice guidelines [70]
Fontan-Associated Liver Disease	Moderate	Starting 10 years post-Fontan, CT/MRI at baseline, ultrasound and AFP every 6 months + CT/MRI every 1–2 years	Expert consensus [37]
Congenital Portosystemic Shunts	Low	Pre-shunt closure: imaging every 6 months Post-shunt closure: imaging every 3 to 6 months for 2 years, and yearly beyond that	Expert consensus [56]
Cavernous transformation of the portal vein	Very low	Consider ultrasound every 6 months in presence of liver disease	No specific guideline
Porto-Sinusoidal Vascular Disorder	Very low	Not currently recommended; consider ultrasound every 6 months for the risk of PVT development	No specific guideline

Abbreviations: AFP, alpha-fetoprotein; CT, computed tomography; MRI, magnetic resonance imaging.

**Table 2:** Type of liver lesions and prevalence of hepatocellular carcinoma (HCC) in the most common vascular liver diseases.

Vascular Liver Diseases	Definition	Types of Hepatic Lesions	HCC Frequency	Pathophysiological Mechanism
BCS 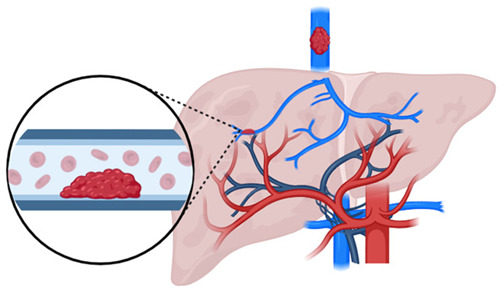	Obstruction of hepatic venous outflow tract (between HVs and the junction IVC and the right atrium)	- NRH - FNH/FNH-like - HCA - HCC	Frequent (cumulative incidence 0.3%, 4.7% and 7.7% after 1-, 3-, and 5-years [14])	Hepatic congestion + centrilobular ischemia → ↑ compensatory arterial inflow + liver cirrhosis
FALD 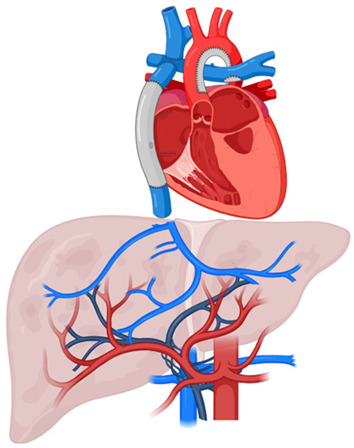	Congestive hepatopathies caused by hemodynamic disturbances following Fontan surgery in patients with univentricular congenital heart disease	- FNH/FNH-like - HCA - HCC	Moderately common (cumulative incidence 0%, 2% and 7% after 10-, 20- and 30 years, respectively, from surgery [40]; annual incidence 1.04% in pts with liver cirrhosis [42])	Chronic increase in central venous pressure + ↓ hepatic drainage → hepatic congestion + hypoxia → ↑ compensatory arterial inflow + liver cirrhosis
CPSSs 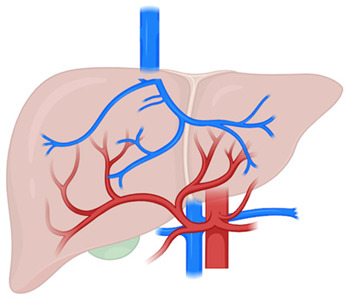	Alterations in the communication between the PV and the systemic circulation	- NRH - FNH/FNH-like - HCA - HCC	Rare	Deprivation of portal inflow → arterial hyperperfusion → proliferative stimulus
CTPV 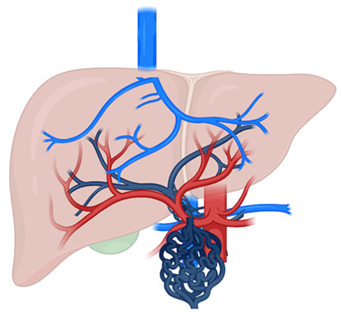	Compensatory response to chronic PVT with collateral vessel formation	- FNH/FNH-like - HCA - HCC	Very rare	Altered portal perfusion → arterial adaptation with increased hepatic arterial flow
PSVD 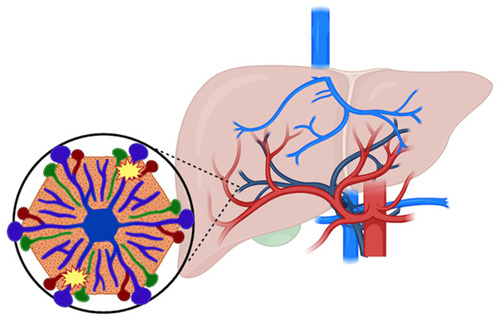	Portal microvascular pathology involving the portal venules and sinusoids in the absence of cirrhosis.	- NRH - FNH/FNH-like - HCA - HCC	Very rare	Portal venules and sinusoids disorder → reduced portal venous inflow + increased compensatory arterial inflow

Abbreviations: BCS, Budd–Chiari Syndrome; HVs, hepatic veins; IVC, inferior vena cava; NRH, nodular regenerative hyperplasia; FNH, focal nodular hyperplasia; HCA, hepatocellular adenoma; HCC, hepatocellular carcinoma; ↑, increased; FALD, Fontan-associated liver disease; patients, pts; ↓, decreased; CPSSs, congenital portosystemic shunts; PV, portal vein; CTPV, cavernous transformation of the portal vein; PVT, portal vein thrombosis; PSVD, porto-sinusoidal vascular disorder.

## Data Availability

No new data were created or analyzed in this study. Data sharing is not applicable to this article.
